# Supplementary material for: Diffusion controls local versus dispersed inheritance of histones during replication and shapes epigenomic architecture
Source: PLoS Comput Biol. 2023 Dec 18;19(12):e1011725. doi: 10.1371/journal.pcbi.1011725 (PMC10760866; doi:10.1371/journal.pcbi.1011725)
Supplement: Attachment — Submitted filename: Response to Reviewers.pdf [file pcbi.1011725.s002.pdf]

<u>Response to Reviewers</u>

Reviewer #1: This is very interesting study which tries to explain systematic difference in the epigenome in more active and less active regions of the chromosomes. It assumes that during replication the histones (more specifically, the tetramer) are not just randomly distributed between the two daughter strands but that they might diffuse to other locations of the daughter strands as well. The assumption is that systematic differences occur since the diffusion constants for the two types of regions differ. In the active regions epigenetic marks become more equal after a few cell divisions than in less active regions. The authors build a simple model to demonstate this and also study real data, finding an astonishing similarity to their predictions. I find this study well written and the results striking. Being a physicist, I would have done the modeling probably different (I give some thoughts about this below) but this is just a matter of background and taste. So I recommend publication of the manuscript after the points mentioned below have been considered and/or clarified.

Response: We thank the reviewer for the summary, very positive comments, and the recommendation for publication. Here we respond to all the clarifications asked for by the reviewer. Also, all the code for the theoretical model and the bioinformatic analyses is now available on https://github.com/Shaonlab/Simulation-DADs and https://github.com/Shaonlab/Diffusion-Accessible-Domains respectively.

1. I find the model a bit unconventional as it combines some rather unphysical representation of the chromosome in a matrix with a real physical process (diffusion). So what are the distances between nucleosome in a row of the matrix in real space? The authors essentially assume 1D diffusion but in real space the histones will more likely diffuse in 3D (with possibly a fraction of time diffusing even in 1D along the DNA). I spontaneouly would think that it would be better to assume that spatial distances vary with the square root of the contour distance along the DNA, as the chromatin fiber forms a random walk, at least for larger distances. But is is not quite clear when this crossover to the random walk takes place and so the current assumption in the model is acceptable for me. The authors are well aware of this and discuss it at the end of the paper but I would suggest to move this discussion close to Eq. 1.

Response: The reviewer is absolutely right in pointing out that diffusion (at least to the larger distances, especially across chromosomes) will likely occur in 3D space as opposed to 1D. But keeping in mind the nature of datasets that primarily exist at the moment (contact frequencies using the HiC technique), we felt that implementing a more accurate 3D model might not provide much added benefits over assuming a simpler 1D diffusion model. Since HiC does not provide information on real distances, best fit diffusion constants from our model cannot be interpreted physically, whether a 1D or 3D model is implemented. Hence we decided to keep the model as simple as possible and stick to a 1D description.

As per the suggestion of the reviewer, we have now added a short discussion of these limitations closer to Equation 1, while retaining it in the Discussion section as well, so that the limitations are not overlooked by the reader.

2. Why are the j=1 nucleomes depicted larger in Fig. 2?

Response: We are sorry that this was not clear from the Figure descriptions. The reason to show the j=1 nucleosomes larger was to visually suggest that only one column of nucleosomes is evicted at a time, and that diffusion and redistribution of these nucleosomes happen *only within the rows of a single column*, not across columns. This step in our model amounts to making an assumption that the replication fork progresses slower than the time taken for histones to diffuse and find their new landing spots in the newly created DNA strands. While we used this rule to simplify our model, it is not a bad assumption and is likely to hold for many genomic loci. The average replication speed in eukaryotes is about 2000 bp/minute, which is 33 bp/s (Mechali, Nat Rev Mol Cell Biol, 2010). At this speed, it will take the fork about 4-5 seconds to reach the next histone 147 bp away. However, it has been observed that histone reassembly behind the fork occurs very rapidly, on the order of seconds (Annunziato, Biochim Biophys Acta, 2012). Therefore diffusion within rows of the same column in our model, though a simplification, is not entirely inaccurate. This point is discussed in detail in the section “Assumptions underlying Model 1” of the Supplementary Information.

We have now added a few sentences to explain this assumption and the reason for depicting larger j = 1 nucleosomes in the main text, Figure 2 legend, and have elaborated on this point in the SI.

3. It would be good to explain the initial conditions used in Fig 3 for the marks in the main text and not only in the legend to that figure. What means "exponential distributions with parameter 130"?

Response: We have 200 histones (200 columns) per row in our model, which we populated with “1”s (representing a certain histone mark of interest, say H3K27me3) to begin our simulations. To assign a random number of “1”s per row, but keeping the average number at 130, we drew 20 random numbers (since we have 20 rows) from an exponential distribution with an average of 130. These numbers were then used to randomly assign “1”s in every row. The remaining unassigned spots were given the label “2”, representing some other histone modification not of our interest. We have checked that the (arbitrary) choice of 130 does not make any qualitative difference to the ultimate patterns that we get from our simulations. This procedure is explained in detail in the Supplementary Information.

We have now clarified this initial condition in the Methods Section of the main text.

4. I did not understand the argument in the legend to Fig. 3 why distances are measured negative. In Hi-C contact maps the distance increases as one moves away from the diagonal. So in the top row of such a map, distances increase to the right, in the bottom row they decrease. So both cases actually occur.

Response: We are sorry for this confusion. From HiC maps we obtain either spatial proximity (which is a Pearson Correlation, explained in detail in our Methods section) or frequency of contacts between two genomic loci. In both cases, increasing values are expected to approximately correspond to *decreasing* physical distance. This is why in our histone mark similarity (Q) versus spatial proximity plots, moving towards the right of the x-axis represents decreasing physical distance of loci pairs. To match this trend in plots generated from our simulations, we therefore plot the negative of the distance on the x-axis. Plotting the negative distance on the x-axis therefore simply helps visually comparing plots generated from real datasets versus those generated from our simulations.

We have now added a clarification in the main text.

5. (not for this paper) I am not sure if it is possible and whether the authors have considered it, but if not they might want to check whether their calculations could be strongly simplified by just solving the diffusion equation and this way simply following the spread of the marks along the 1D chain.

Response: We thank the reviewer for this interesting suggestion. Indeed, it might be possible that this problem is at least approximately but analytically soluble, in particular if it is formulated using transition rates and a Master Equation framework. Approaches like this (together with transfer matrices to describe cell division) have been used in the past to investigate the origins of bistability in epigenetic landscapes (Micheelsen et al, Physical Biology 2010), and would be an interesting way to reformulate our problem. We will think along these lines in future studies on this problem.

Reviewer #2: The authors proposed a stochastic model of histone diffusion and spreading of histone marks upon replication, motivated by an experimental report on the histone diffusion, and examined the model via bioinformatic analysis using independently available set of genomic experimental data (Hi-C, replication timing, and ChiP-seq for epigenetic marks). While the model is new, sound, and potentially valuable, I consider the verification is rather weak and thus cannot judge if the model is indeed validated or not. I need at least major update before making a clear judgment.

Response: We thank the reviewer for the concise summary and for appreciating the originality of our modelling framework. We agree that there were some important limitations of our manuscript, and have performed a range of new analyses to address the concerns raised by the reviewer. Point by point responses are provided below. Also, all the code for the theoretical model and the bioinformatic analyses is now available on https://github.com/Shaonlab/Simulation-DADs and https://github.com/Shaonlab/Diffusion-Accessible-Domains respectively.

1) My major concern is the critical difference between the stochastic model and the bioinformatic verification. In the former, the distance can represent the physical distance. In the latter, it is the HiC distance defined by the author, which can be even qualitatively different from the physical difference. There is no clear argument of the similarity of the two "distances".

Response: We agree with the reviewer that there is indeed a difference between the physical distance in our model and the spatial proximity metric from the HiC data, which we had pointed out as a limitation in our Discussion section. As the reviewer correctly points out, it is indeed not necessary that there be a one-to-one relationship between physical distances and HiC measures of proximity, which measure frequency of contacts. Having said that, it is important to note that often however there *is* a strong relationship or correlation – *larger the frequency of contacts between two genomic loci, the more likely that the two loci are at a shorter physical distance*. This was pointed out in the original HiC paper, where a strong negative correlation was observed between the HiC read count and average distance (Fig. S3 of Lieberman-Eiden et al, Science 2009). In subsequent analyses, the majority of genomic loci that have been investigated using methods like DNA-FISH, have been demonstrated to follow this trend (Rao et al, Cell 2014; Fudenberg and Imakaev, Nat Meth 2017). Even in cases where this relationship does not hold and larger physical distances correspond to higher HiC contact frequency, this phenomenon only seems to occur at large length-scales, at distances greater than ~ 0.6 microns (Fig. 2b of Fudenberg and Imakaev, Nat Meth 2017). Below these distances the expected relationship between HiC contact frequency and physical distance still holds. Therefore, as originally pointed out in Lieberman-Eiden et al, and verified later by others, HiC read counts usually serve as a good proxy for distance. Therefore our model, while not perfect, should be sufficient for comparison with HiC data. When more genome-wide DNA-FISH datasets becomes available in the future, more direct comparisons of data would become possible using our model.

We have now added these details in our Discussion section and the SI. We have also added some new analyses using variations of the HiC distance measure, as explained in the next point.

1a) Related to this point, the HiC distance is not defined clearly. At least, I did not find the definition. This is one of the most important quantity and thus the use of it without the clear definition is critical.

Response: We apologize for not clearly defining the HiC distance in the manuscript. This measure which we called "Spatial Proximity" following Khrameeva et al, Plos One, 2012, is actually a Pearson Correlation that was introduced in the original HiC paper Lieberman-Eiden et al, Science 2009. To generate this correlation, a normalized contact matrix M^* is defined by dividing each entry in the contact matrix by the genome-wide average contact probability for loci at that genomic distance (Lieberman-Eiden et al, Science 2009). Then the correlation matrix C is defined in which c_{ij} is the Pearson correlation between the ith row and j^{th} column of M^*. The physical reasoning used in the original paper to use this correlation matrix as a measure of spatial proximity is that if two loci (1 Mb regions in our work) are nearby in space, they will share neighbours and have correlated interaction profiles. The authors went on to demonstrate that using the correlation matrix instead of the contact matrix dramatically sharpened the plaid pattern that is characteristic of HiC maps (Fig. 3A-C of Lieberman-Eiden et al, Science 2009).

To further address the reviewer's concern regarding the choice of the HiC distance metric, we carried out a series of new bioinformatic analyses. Instead of using just the Correlation matrix (spatial proximity) as a measure of physical distance, we additionally looked at Observed and Observed/Expected HiC counts. We analyzed data from both cell types GM06990 and K562, and plotted the histone mark similarity Q(i,j) as functions of the Observed and Observed/Expected HiC counts. In both cases we found patterns largely consistent with predictions of our modelling, thereby demonstrating that our results are not spuriously arising from use of one particular metric of HiC distance. The patterns were however not as clear as compared to using the Correlation matrix, but this was to be expected from Lieberman-Eiden et al, Science 2009, where they demonstrated that using the Correlation matrix helps reduce noise and sharpen the plaid patterns typical of HiC maps.

We have now added the definition of Spatial Proximity in our main text, Figure 4 legend and also added a short discussion of the new analyses, along with a new **Supplementary Figure 7**.

2) The authors call their model, the DAD model meaning the "diffusion-accessible-domain". Surprisingly, even the DAD is not clearly defined in the stochastic model of histone diffusion.

Response: Please see our response to (2a) below.

2a) Can the authors estimate the typical size of the DAD from the bioinformatic analysis of human genome, once the DAD is clearly defined.

Response: We are envisioning these "Diffusion-Accessible-Domains" or DADs to be spatial domains centred around each histone, accessible with non-zero probability via diffusion. The size of these domains will vary since diffusion constants are different according to whether the histone is within the euchromatin or heterochromatin environment. Unfortunately it is challenging to estimate the size of these domains since accurate quantification of the diffusion constants in different genomic contexts is not easily available, and neither are measurements of the time-scales of diffusion. Scouring the literature, we managed to make a very rough upper-limit estimate of $R = \sqrt{6\,D\,t} \approx 8\,\mu m$ using the following estimates: (1) $D \sim 10\ \mu m^2/s$ (Bhattacharya et al, Biophys J 2006; Mangenot et al, Biophys J, 2003) and (2) Diffusion time t on the order of seconds before the nucleosomes get reassembled behind the replication fork (Annunziato, Biochim Biophys Acta, 2012). This estimate of $8\,\mu m$ would be consistent with our results that diffusion occasionally transfers histones from one chromosome to another, since distances between chromosomes are of the order of 3-5 μm (Heride et al, J Cell Science, 2010). These estimates are however currently not possible from the bioinformatic analyses that we have performed, mainly because the HiC data does not provide real distances. Current attempts in the field at inferring real distances from HiC contact maps will hopefully allow such estimates to be made in the future.

We have now included these estimates in the main results section introducing the DAD hypothesis and also discussed the limitation in the Discussion section.

3) If I understood correctly, in the model 1, the histone diffuses to different loci (different row in the matrix), but not the different nucleosome (column of the matrix). I think it odd since neighboring nucleosomes are much closer than the neighboring genomic loci. If this is right, authors need to explain the idea behind this modeling. I might have misunderstood the model. If so, the authors need to make the description clearer.

Response: The Reviewer is absolutely correct regarding this aspect of our Model. While we had explained this point in the "Assumptions underlying Model 1" section of the SI, we apologize for not explaining this better in the main text. Allowing only "vertical" (within rows of the same column) but not "horizontal" (columns of the same row) diffusion amounts to assuming that histone diffusion and subsequent capture by a genomic locus occurs faster than the time it takes for the replication fork to move ahead and displace the next histone. While we used this rule to simplify our model, it is not a bad assumption and is likely to hold for many genomic loci. The average replication speed in eukaryotes is about 2000 bp/minute, which is 33 bp/s (Mechali, Nat Rev Mol Cell Biol, 2010). At this speed, it will take the fork about 4-5 seconds to reach the next histone 147 bp away. However, it has been observed that histone reassembly behind the fork occurs very rapidly, on the order of seconds (Annunziato, Biochim Biophys Acta, 2012). Therefore disallowing "horizontal" diffusion, while an approximation, is unlikely to be a severe limitation of the model. Furthermore, as we estimated in the point above, even in a couple of seconds a histone component can diffuse up to ~ 8 microns, sufficient to reach a neighboring genomic locus.

We have now provided more details along these lines and the estimates in the main text as well as the SI.

4) I thought the equation (2) is odd. Possibly, the placement of the vertical bars (absolute value) is wrong. The vertical bars for the entire log makes more sense, to me.

Response: We thank the reviewer for picking up this typo. We have now corrected it.

5) As a minor comment, the notations for the matrix are somewhat misleading, at least to me. In the definition of the DAD model, j represents the column of the P, or D matrix meaning the nucleosomes. The j is used to represent genomic locus. It would be much better to use the same symbols for the same meaning throughout the paper.

Response: We thank the reviewer for carefully reading the manuscript – we agree, usage of "j" to represent genomic loci in Model 2 and the Bioinformatic analysis (especially in defining the similarity *Q(i,j)*) can indeed be very confusing, since we used "j" to denote positions of the nucleosomes in Model 1.

We have now changed all the *Q(i,j)*s, including in the Figures, to read $Q(i_1, i_2)$, to clarify that the similarity is being calculated between two genomic loci i_1 and i_2.
